# The impact of cytochrome 450 and Paraoxonase polymorphisms on clopidogrel resistance and major adverse cardiac events in coronary heart disease patients after percutaneous coronary intervention

**DOI:** 10.1186/s40360-019-0378-7

**Published:** 2020-01-03

**Authors:** Zhaowei Zhang, Mingxiao Chen, Long Zhang, Qiang Zhao

**Affiliations:** 1 Department of Pharmacy, Jin Hua Municipal Central Hospital, Jin Hua, 32100 China; 2 Department of Medical laboratory, Jin Hua Municipal Central Hospital, Jin Hua, 32100 China; 3 Department of Vascularcardiology, Jin Hua Municipal Central Hospital, Jin Hua, 32100 China

**Keywords:** Cytochrome 450, Paraoxonase-1, Polymorphism, Clopidogrel resistance, Major adverse cardiac events

## Abstract

**Background:**

Clopidogrel is an inactive prodrug, it catalyzed into its active form by Cytochrome 450 and Paraoxonase-1(PON-1). polymorphisms of genes encoding these enzymes will affect the efficacy of Clopidogrel. The main objective of our study was to investigate the association of CYP2C19*2, CYP2C19*3 and PON-1Q192R polymorphisms with Clopidogrel resistance and major adverse cardiac events in Jin Hua district in the middle of Zhe Jiang Province in China.

**Methods:**

One hundred sixty coronary heart disease patients with percutaneous coronary intervention, who were followed-up for 1 year, were enrolled in our study. These patients were co-administered aspirin 100 mg/d and clopidogrel 75 mg/d following a loading dose of 300 mg. The ADP-induced platelet aggregation rate was measured by Platelet aggregator. Genotypes of CYP2C19*2, CYP2C19*3, PON-1Q192R were determined using Sanger sequencing in all patients. Various clinical data were collected.

**Results:**

The frequencies of CYP2C19*2, CYP2C19*3 and PON-1Q192R homozygous mutant genotypes were significantly lower in non-responders than those in responders. After for all variables, CYP2C19*2, CYP2C19*3 and PON-1Q192R independently increased the risk of clopidogrel resistance with adjusted ORs 46.65(95% CI,1.77–25.04; *p* = 0.005); 22.74(95% CI, 3.11–166.27; *p* = 0.002); 5.69 (95% CI,1.06–30.47; *p* = 0.042). Over a follow-up of 12 months, the incidence of major adverse cardiac events (MACE) in CYP2C19*1/*2, *1/*3, *2/*2, *2/*3 was significantly higher than no mutant genotype (18/40vs.2/63,3/9vs.2/63, 11/6vs.2/63, 7/1vs2/63, respectively). There was no significant correlation between PON-1Q192R mutant allele and MACE.

**Conclusion:**

Our study was first time to report on CYP2C19 and PON-1 polymorphisms in Jin Hua population in the middle of Zhe Jiang province in China. The carriage of CYP2C19*2 or *3 mutant allele significantly reduced the platelet response to clopidogrel and increase the MACE. The carriage of PON-1 mutant allele also significantly reduced the platelet response to clopidogrel, but would not increase the major adverse cardiac events after 1 year follow-up.

**Trial registration:**

ChiCTR, ChiCTR1800018316. Registered 11 September 2018 – prospective registered, http://www.chictr.org.cn/edit.aspx?pid=30927&htm=4.

## Background

Coronary Heart Disease (CHD) is a significant cause of morbidity and mortality in the world [1]. Percutaneous Coronary Intervention (PCI) is the principal revascularization strategy employed in the treatment of CHD [2]. Dual antiplatelet therapy (DAPT), with aspirin and Clopidogrel, is a basis for the care of patients after percutaneous coronary intervention (PCI) [3]. Despite adequate antiplatelet therapy, up to 10% of patients will still experience a recurrent acute ischemic event, this is probably because of significant interindividual variability in the clopidogrel response [4, 5] .

Clopidogrel is an inactive prodrug. The absorption of Clopidogrel is mainly limited by P Glycol-.

protein (P-gp) which is encoded by ABCB1.Then it undergoes 2 sequential oxidative stages in order to be active. Firstly, clopidogrel catalyzed by Cytochrome 450 (CYP2C19, CYP2B6, CYP1A2) into 2-oxo-clopidogrel, then CYP3A4, CYP2C9 and the Paraoxonase (PON-1) enzyme transform 2-oxo-clopidogrel into its active form [6–8].It has been confirmed that CYP2C19 is the most important enzyme involved in clopidogrel response. Whereas genetic polymorphism of CYP2B6, CYP3A4, CYP2C9 showed minor effects on clopidogrel response [9]. Here, we mainly studied the association between PON-1Q192R, CYP2C19*2, CYP2C19*3 allelic variants and clopidogrel response. Several studies have been confirmed that carriers of the allelic variant (CYP2C19*2) is correlated with adverse cardiovascular events and low clopidogrel response [10, 11]. whereas studies about the relevance between the allelic variant of CYP2C19*3, PON-1 Q192R and the clopidogrel response are discrepancy [12–15]. Moreover, the polymorphism of CYP2C19 and PON-1 varies considerably with both geographical location and ethnic group [16]_._In addition, To our knowledge, there are no studies about the relationship between genetic polymorphisms and clopidogrel resistance among the CHD patients in Jin Hua in the middle of Zhe Jiang province in China. The aim of our study was to determine the association between the allelic variant of CYP2C19*2, CYP2C19*3, PON-1 Q192R and clopidogrel resistance and major adverse cardiac events (MACE) among CHD patients in the middle of Zhe Jiang province in China.

## Methods

### Study population

This study was approved by the Ethics Committee of Zhe Jiang Provincial Jin Hua Municipal Central Hospital, China. 160 Chinese Han patients who were born in Jin Hua and diagnosed as CHD scheduling to undergo PCI were recruited into our study. We have calculated the sample size before the study by Quanto software. Power calculation was performed by Quanto software. Assuming a allele frequency of 0.33 and disease prevalence of 5–26%, we had 80% power to detect genetic effects at an OR of 2.25 under an additive model in our samples. All the patients took 300 mg clopidogrel and 300 mg Aspirin before PCI, then they persistent on 75 mg/day clopidogrel and 100 mg Aspirin for 12 months. The inclusion criteria were: (1) Chinese Han CHD patients undergoing PCI who was born in Jin Hua (2) patients who never took clopidogrel before enrolled. The exclusion criteria were (1) patients who were allergic with Clopidogrel and Aspirin; (2) patients who experienced the bleeding and cerebrovascular disease before the last 6 months; (3) patients with severe renal and hepatic function failure;(4) patients who can live less than 12 months;(5)patients with large surgery within 1 month.

### DNA extraction

We collect the blood samples in EDTA tubes and stored at − 80 °C. The extraction procession was following the manufacturer’s recommendations of the whole blood genomic DNA extraction kit (Tiangen biotech company, Bei Jing, China).

### Platelet aggregation assays

Blood samples for platelet function testing were done before and 7 days after PCI. Blood samples(3 ml) were collected in the tube containing 10 [9] μmol/L sodium citrate. The platelet-rich plasma (PRP) and the platelet-poor plasma (PPP) were separated by centrifuged. The PPP and PRP samples were heated to 37 °C for 3 min, then 20 μmol/L of ADP was added to the samples to measure platelet aggregation rate (PAR). (PAR _before_-PAR _after_)/ PAR _before_ was used to calculate the platelet aggregation inhibition rate (PAIR). When PAIR is less than 10%, we considered these patients as clopidogrel resistance.

### Genotyping assay

Genotyping of the CYP2C19*2, CYP2C19*3 and PON-1Q192R were accomplished by polymerase chain reaction (PCR). The primer sequences for PCR were as below: for CYP2C19*2, forward primer 5`-ACCAGAGCTTGGCATATTGTATCT-3`, reverse primer 5`-GATTCTTGGTGTTCTTTTACTTTCT-3`, 192 bp; for CYP2C19*3, forward primer 5`- TTTCATCCTGGGCTGTGCTC-3`reverse primer 5`-TGTACTTCAGGGCTTGGTCAAT-3`, 234 bp; for PON-1, forward primer 5`-AAGGCTCCATCCCACATCT-3’and reverse primer 5`- CATCGGGTGAAATGTTGATT − 3`, 312 bp. PCR amplification was done by a S1000 Peltier thermal cycler (Biorad, USA) in the following conditions: Firstly, denaturing at 94 °C for 5 min, followed by 35 cycles including at 94 °C about 30 s, at 60 °C (CYP2C19*2), 54 °C (CYP2C19*3), 58 °C (PON-1Q192R) for 30s, 72 °C for 30s. Finally, extending at 72 °C for 10 min. All these PCR products were sequenced (Hangzhou Qing ke Biotechnology Co., Ltd., Hangzhou, China). According to the CYP2C19 genotype, these patients were categorized into three groups: normal metabolizers (*1/*1), intermediated metabolizers (*1/*2, *1/*3) and poor metabolizers (*2/*2, *2/*3, *3/*3). On the basis of the PON-1Q192R genotype, these patients also could be divided into QQ(AA) genotype, QR(AG) genotype, RR(GG) genotype.

### Follow-up

Patients were followed up at the 6, 12 months after PCI. The incidence of recurrent angina, nonfatal MI, cerebral stroke, stent thrombosis, and sudden cardiac death were recorded as the main endpoints. The patients were consulted face to face or by telephone.

### Statistical analyses

The deviation between genotype distribution and Hardy-Weinberg equilibrium (HWE) was analyzed by the Chi-square test. The linkage disequilibrium(LD) analysis was performed by Haploview and the haplotypes analysis was conducted by SHEsis (available on line http://analysis.bio-x.cn/myAnalysis.php). Mean ± SD was used to express the continuous data, the categorical data presented as percentage. The comparisons among different groups were performed by one way analysis of variance (ANOVA). Whereas, Chi square and Fisher’s exact test were suitable for the categorical variable. *p* < 0.05 means there are statistically significant among different groups. Both Univariable and multivariable logistic regression analyses were used to analysis the relationship between the genotypes of PON1 Q192R, CYP2C19*2 CYP2C19 *3 and clopidogrel resistance. All these analyses were using SPSS 23.

## Results

### Genotype results of CYP2C19 and PON-1

**Fig. 1:**
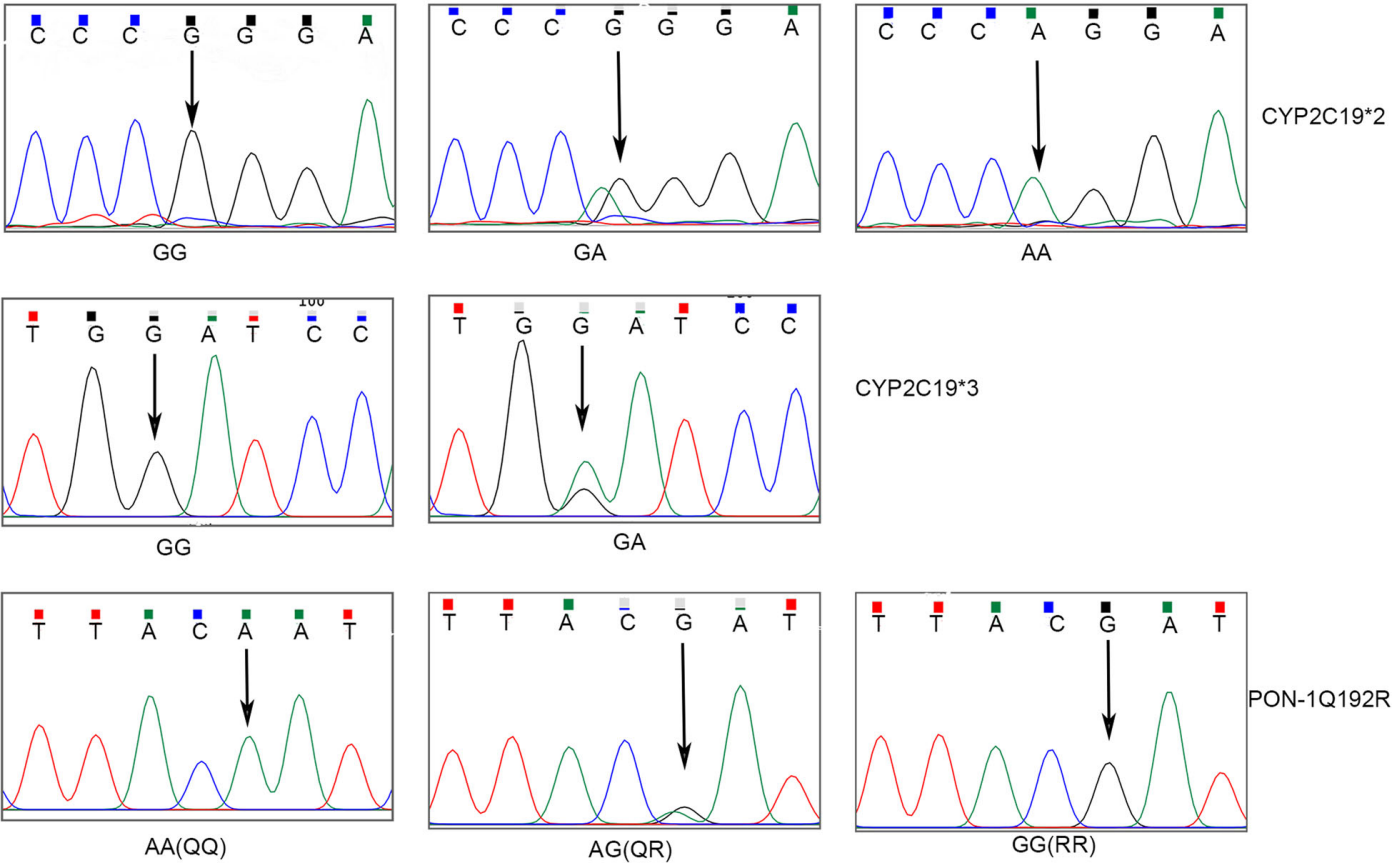
Sequence of CYP2C19*2, CYP2C19*3 and PON-1Q192R. Sequence chromatogram of CYP2C19*2, CYP2C19*3 and PON-1Q192R. SNPs are indicated by arrows

CYP2C19 and PON-1 genotypes were assayed in 160 patients. The distribution of CYP2C19*2, CYP2C19*3, PON Q192R allelic and genotype frequency are shown in Table 1, Fig. 1. It seems no significantly difference between our results and the Hardy-Weinberg equilibrium (*p* = 0.553, *p* = 0.152 and *p* = 0.4, respectively).

**Table 1:** Genotype distribution and allele frequency of CYP2C19*2、*3、PONQ192R

	genotype(n/%)		Allele frequency(n/%)		HWE	
	GG	GA	AA	G	A	
CYP2C19*2	78(48.75)	65(40.62)	17(10.63)	221(69.06)	99(30.94)	0.533
CYP2C19*3	140(87.5)	20(12.5)	0(0)	300(93.75)	20(6.25)	0.152
PONQ192R	73(45.62)	64(40)	23(14.38)	210(65.62)	110(34.38)	0.4

*p* < 0.05 was considered significant. *HWE* Hardy-Weinberg equilibrium

### Baseline characteristics of participants

All the enrolled patients were divided into clopidogrel-resistant group(*n* = 40) (PAIR≤10%) and clopidogrel-responded group(*n* = 120) (PAIR≥10%). The baseline characteristics of these two groups were concluded in Table 2. The average age of these patients were 62.35 ± 12.76 years(male vs female: 75.63% vs 24.37%). Among these patients, almost 70% of the patients presented with acute myocardial infarction (AMI). Most of the patients (91.25%) were intervened with drug-eluting stents. The baseline of demographics, clinical presentations and drug combinations were similar between the clopidogrel responded group and the clopidogrel-resistant group (*p* > 0.05) except for the incidence of diabetes and smoking(*P* < 0.05). The genotype distribution of CYP2C19*2, *3 and PON1 Q192R among these two groups were showed in Table 3, No mutant homozygous of CYP2C19*3 genotype was found in our study. The proportion of CYP2C19*1/*1 genotype frequency was obviously higher in clopidogrel responded group (50.83% vs 10%). While the frequency of CYP2C19 (*2/*2, *2/*3) were higher in the clopidogrel-resistant group (27.5% vs 5,17.5% vs 0.83%). Meanwhile the proportion of patients with PON-1Q192R(GG) genotype were higher in clopidogrel resistant group than those in clopidogrel response group(67.5% vs 38.33%).

**Table 2:** Baseline characteristics of study patients

Parameters	Total (*n* = 160)	Non-responders (*n* = 40)	Responders (*n* = 120)	*P*
Age	62.35 ± 12.76	60.83 ± 11.47	62.86 ± 13.17	0.385
Male/Female(n/n)	121/39	31/9	90/30	0.834
BMI	24.03 ± 3.06	24.09 ± 3.47	24.01 ± 2.92	0.881
Hypertension(%)	108(67.5%)	24(60%)	84(70%)	0.165
Diabetes(%)	40(25%)	17(42.5%)	23(19.17%)	0.003*
smoking(%)	62(38.75%)	21(52.5%)	41(34.17%)	0.039*
Dyslipidemia(%)	148(92.5%)	36(92.31%)	112(93.33%)	0.732
ACEI/ARB (%)	81(50.63%)	19(47.5%)	62(51.67%)	0.716
CCB(%)	38(23.75%)	9(22.5%)	29(24.17%)	0.508
Statin(%)	152(95%)	36(92.31%)	116(96.67%)	0.2
PPI (%)	48(30%)	14(35%)	34(28.5%)	0.272
Nitrates	65(40.625%)	18(45%)	47(39.17%)	0.515
β-receptor blocker	148(92.5%)	36(90%)	112(93.33%)	0.488
Diuretics	22(13.75%)	6(15%)	16(13.33%)	0.791
Rivaroxaban	12 (7.5%)	4(10%)	8(6.67%)	0.488
Panax Notoginseng	14(8.75%)	5(8%)	9(7.5%)	0.332
Metal stent	14 (8.75%)	3(7.5%)	11(9.17%)	0.747
Drug-eluting stent	146(91.25%)	37(92.5%)	109(90.83%)	0.747
Infarct artery LAD	115(71.88%)	29(72.5%)	86(71.67%)	0.919
Infarct artery LCX	108(67.5%)	24(60%)	84(70%)	0.242
Infarct artery RCA	135(84.38%)	34(85%)	101(84.17%)	0.9
Infarct artery LM	35(21.88%)	8(20%)	27(22.5%)	0.74
clinical presentation				
Angina	25(15.63%)	6(15%)	19(15.83%)	0.9
AMI	116(72.5%)	27(67.5%)	89(74.16%)	0.413

*CCB* Calcium channel blockers

*PPI* Proton pump inhibitors

*ACEI/ARB* angiotensin converting enzyme inhibitor/ angiotensin II receptor antagonist

*LAD* left anterior descending artery

*LCX* left circumflex artery

*RCA* right coronary artery

*LM* left main coronary artery

*AMI* acute myocardial infarction

*Variable is significant difference between responders and non-responders at *p*-value< 0.05

**Table 3:** Distribution of CYP2C19*2, *3 and PON1 genotypes in clopidogrel responder and non-responder group

	Genotype	Non-responders (40)	responders (120)	*P*
CYP2C19	^a^1/^a^1(681GG/636GG)	4(10%)	61(50.83%)	< 0.001
(^a^2:681G > A	^a^1/^a^2(681GA/636GG)	13(32.5%)	45(37.5%)	0.569
^a^3:636G > A)	^a^1/^a^3(681GG/681GA)	5(8%)	7(5.83%)	0.177
	^a^2/^a^2(681AA/636GG)	11(27.5%)	6(5%)	< 0.001
	^a^2/^a^3(681GA/636GA)	7(17.5%)	1(0.83%)	< 0.001
	^a^3/^a^3(681GG/636AA)	0	0	
PONQ192R	AA(QQ)	2(5%)	21(17.5%)	0.172
(575A > G)				
	AG(QR)	11(27.5%)	53(44.17%)	0.066
	GG(RR)	27(67.5%)	46(38.33%)	0.001

^a^ Variable is significant difference between responders and non-responders at *p*-value< 0.05

### CYP2C19*2, *3 and PON1 Q192R genotypes and platelet aggregation inhibition rate

**Fig. 2:**
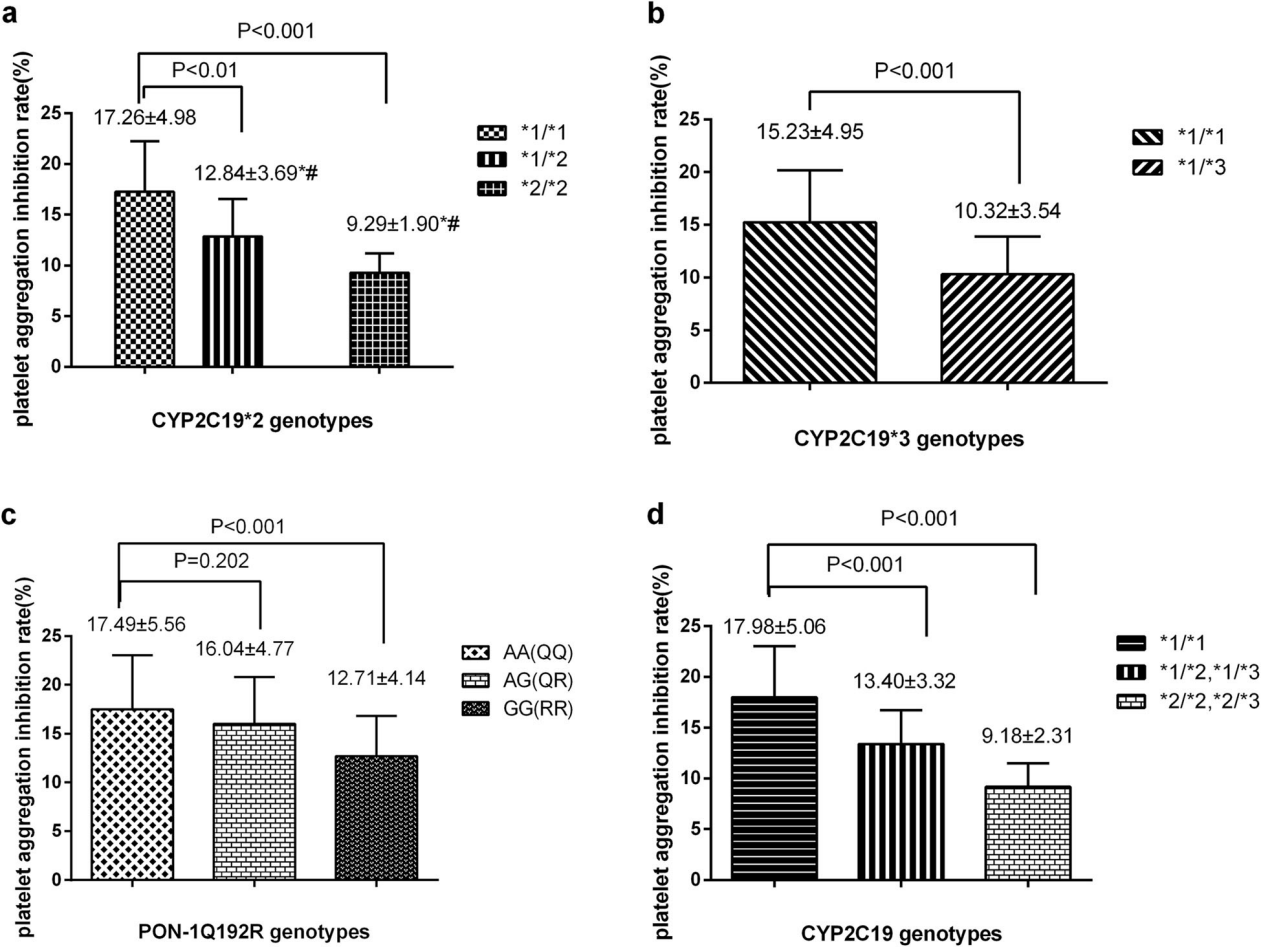
CYP2C19*2, CYP2C19 *3, PON-1Q192R genotypes and ADP- induced platelet aggregation inhibition rates. **a**: CYP2C19*2 and ADP induced platelet aggregation inhibition rates; **b**: CYP2C19*3 and ADP induced platelet aggregation inhibition rates; **c**: PONQ192R and ADP induced platelet ggregation inhibition rates; **d**: CYP2C19*2 or *3 genotypes and ADP- induced platelet inhibition rates; *p*-value< 0.05: ADP induced platelet aggregation inhibition rates is significant difference between different genotype

The platelet aggregation inhibition rate (PAIR) in all the CYP2C19*2, *3, PONQ192R genotypes were showed in Fig. 2. The PAIR of the patients with the genotype of CYP2C19 (*1/*2 or *2/*2) were significantly lower than those in patients with CYP2C19 (*1/*1) (*p* = 0.003, *p* = 0.001). The same results were found in CYP2C19*3 genotypes. When consideration the allelic of both CYP2C19*2 and CYP2C19*3, The PAIR of intermediated metabolizers (*1/*2, *1/*3) and poor metabolizers (*2/*2, *2/*3) were lower than normal metabolizers (*1/*1) (*p* < 0.05). For PON1 Q192R genotypes, the PAIR of patients with RR(GG) genotype were obviously lower than those in patients with QQ(AA) genotype (*p* < 0.05)**.**

### Relationship between CYP2C19 *2, CYP2C19*3, PON1 Q192R genotype and clopidogrel responses

Both univariate logistic regression and the multivariate logistic regression analysis were used to determine the risk factors of clopidogrel resistance. The results were shown in Table 4. The univariate logistic regression showed that CYP2C19*2 (*1/*2) and CYP2C19 (*2/*2) were separately 2.81 times (95% CI,1.12–6.59; *p* = 0.03) and 12.47 times (95%CI, 3.47–41.22; *p* = 0.001) higher risk of clopidogrel resistance. Meanwhile, CYP2C19*3 (*1/*3) predicted the risk of clopidogrel resistance (OR 5.31,95% CI,1.96–14.41; *p* < 0.001). Due to the combined effect of CYP2C19*2 and *3, the *2/*2 and*2/*3 genotypes showed more higher risk of clopidogrel resistances (OR 27.96, 95%CI, 6.77–115.52; *p* < 0.001; OR 106.75, 95%CI,10.42–1093.57; *p* < 0.001).For PON1 Q192R, PON1 Q192R (RR) showed higher risk of clopidogrel resistance (OR 5.47,95% CI,1.19–25.23; *p* = 0.029).

**Table 4:** Association between CYP2C19*2, *3, PON1 Q192R and clopidogrel resistance

Genotype	Crude OR(95%CL)	*P* value	Adjusted OR (95%CL)	*P* value
CYP2C19*2(GG)	1		1	
CYP2C19*2(GA)	2.81(1.12–6.59)	0.018	2.99(1.11–8.03)	0.03
CYP2C19*2(AA)	12.47(3.77–41.22)	< 0.001	8.62(2.29–32.46)	0.001
CYP2C19*3(GG)	1		1	
CYP2C19*3(GA)	5.31(1.96–14.41)	< 0.001	3.64(0.98–13.56)	0.054
1*/1*	1		1	
1*/2*	4.41(1.35–14.41)	0.14	6.65(1.77–25.04)	0.005
1*/3*	10.89(2.36–50.30)	0.002	22.74(3.11–166.27)	0.002
2*/2*	27.96(6.77–115.52)	< 0.001	48.49(8.16–287.97)	< 0.001
2*/3*	106.75(10.42–1093.57)	< 0.001	241.32(14.59–3991.37)	< 0.001
PON1(Q192R)QQ(AA)	1		1	
PON1(Q192R)QR(AG)	2.68(0.56–12.90)	0.22	3.01(0.53–17.05)	0.213
PON1(Q192R)RR(GG)	5.47(1.19–25.23)	0.029	5.69(1.06–30.47)	0.042

*p* < 0.05: Risk is statistical significant when compared to the reference genotype

Multivariable logistic regression analysis was done after adjusting age, sex, BMI, hypertension, hyperlipidemia, type 2-DM, smoking factors. CYP2C19*2 (*1/*2, *2/*2) showed higher risk of clopidogrel resistance (OR 2.99, 95%CI, 1.11–8.03; *p* = 0.03; OR 8.62, 95%CI, 2.29–32.46; *p* = 0.001). Considered the combined effects of CYP2C19*2 and *3, CYP2C19(*1/*2, *1/*3, *2/*2,*2/*3) conferred higher risk of clopidogrel resistance than CYP2C19(*1/*1).While, the PON1 Q192R genotypes (RR) was obviously associate with clopidogrel resistance(OR 5.69,95% CI,1.06–30.47; *p* = 0.042).

### LD analysis and haplotype

The LD analysis showed the LD was observed between rs4986893 and rs4244285(*r* [2] = 0.03). The D` value between rs4986893 and rs4244285 was 0.995. So the haplotype analysis for rs4986893 and rs4244285 was done by the SHEsis. The results of haplotype analysis of the two single nucleotide polymorphisms (SNPs) were showed in Table 5. The AG* and GA* haplotypes were associated with a significant increase in the clopidogrel resistance (OR = 5.119, 95%CL = 2.01–13.035, *P* = 0.000191; OR = 3.299, 95%CL = 1.994–5.597, *P* = 5.8*e-006).

**Table 5:** Haplotype analysis on association of CYP2C19, PON-1 gene and clopidogrel resistance

Haplotypes (rs4244285, rs s4986893)	Frequencies		OR (95% CL)	*P*-values
	Case group N (%)	Control group N (%)		
AG*	12(0.150)	8(0.033)	5.119(2.01–13.035)	0.000191
GA*	41(0.512)	58(0.242)	3.299(1.944–5.597)	5.8e-006
GG*	27(0.338)	174(0.725)	0.193(0.112–0.333)	5.71e-010

The frequency of haplotype below 0.03 was not included in the table; *P*-value < 0.05 was defined as the level of significance

### Clinical outcomes

12 months follow-up were done in all patients. The incidence of major adverse cardiac events(MACE) during the follow-up time was 25.63%.The incidence of MACE occurred less often in CYP2C19(1*/1*) patients than in CYP2C19 (1*/2*, 1*/3*, 2*/2*, 2*/3*) patients (2/63vs.18/40, 2/63vs.3/9, 2/63vs.11/6, 2/63vs.7/1,respectively, *p* < 0.05). While the incidence of MACE occurred in PON1(QQ) patients and PON1(QR + RR) patients were no significantly difference (*p* < 0.05). The results were showed in Table 6.

**Table 6:** Occurrence of adverse cardiovascular events between the different CYP2C19 genotypes and PON-1 Q192R genotypes

group	Genotype	MACE	NON-MACE	*P*
CYP2C19	*1/*1	2	63	
	*1/*2	18	40	< 0.001
	*1/*3	3	9	0.025
	*2/*2	11	6	< 0.001
	*2/*3	7	1	< 0.001
	AA(QQ)	3	20	
PON-1	AG(QR)	13	51	0.534
	GG(RR)	25	48	0.051

*p* < 0.05 was considered significant

## Discussion

Clopidogrel is a prodrug, it needs to be metabolized into an active metabolite with the activity of cytochrome P450 and paraoxonase to playing the role of antiplatelet aggregation^(9).^ The variants of cytochrome P450 and paraoxonase can lead to the change of enzyme activity, especially the mutation of CYP2C19*2, CYP2C19*3, PONQ192R allelic [17, 18]. The probe drug could be used to measure the activity of CYP450 and to point out the genotyping of CYP450 [19]. Ersin Gumus etal evaluate lansopranzole as an in vivo phenotyping probe for assessing CYP2C19 activity, they found that the poor metabolizers have lower enzyme activity [20]. Therefore, the mutation of CYP2C19*2, CYP2C19*3, PONQ192R may cause different clopidogrel response and increased rates of thrombotic events. Although several studies have reported the relationship between CYP2C19*2, CYP2C19*3 and Clopidogrel resistance. Whereas allelic variants exhibit ethnic and geographic diversity [8, 10]. It was the first time for us to assess the relationship between CYP2C19*2, CYP2C19*3, PONQ192R allelic variants and Clopidogrel resistance and to clarify the relationship between the CYP2C19*2, CYP2C19*3, PONQ192R polymorphisms and the incidence of major adverse cardiovascular events in Jin Hua population (in the middle of Zhe Jiang province in China). The results of our study showed that the frequencies of heterozygous and homozygous CYP2C19*2 were 40.62% and 10.63% respectively and the frequencies of heterozygous and homozygous CYP2C19*3 was 12.5, 0%. All genotype frequencies for both CYP2C19*2 and CYP2C19*3 alleles were consistent with Hardy-Weinberg equilibrium. The mutation of CYP2C19*2 is similarly with the results of Wei et al .[21] reported but lower than the results of Zhao et al. [22] reported. While the mutation of CYP2C19*3 showed higher than the results of Zhao et al. reported. Our study also showed that the frequencies of heterozygous and homozygous PONQ192R were 40% and 45.62%, respectively, It is consistent with the results of Xiao-Fang Tang at el [23]reported.

Our study found that both the carriers of CYP2C19*2 and CYP2C19*3 mutant allele showed attenuated response to clopidogrel therapy, after adjusted for age, sex, BMI, hypertension, hyperlipidemia, type 2-DM,smoking, we also found a synergic effect between the two alleles mutation on Clopidogrel resistance, both intermediated metabolizer (*1/*2,*1/*3)and poor metabolizers(*2/*2,*2/*3) all increased the Clopidogrel resistance as compare with normal metabolizers(*1/*1). Our study is consistence with The results of Studies in Thailand [24], Japan [25], Latin American [26], Korea [27]. While Hassani Idrissi [28] et al. showed that a synergic effect between CYP2C19*2 and CYP2C19*3 loss-of-function is associated with Clopidogrel resistance but not CYP2C19*2 and CYP2C19*3 loss-of-function lonely. This may due to inter-racial differences and small sample size. We conducted the haplotype analysis for rs4986893 and rs4244285. We found that the haplotype AG* and GA* were associated with an increased Clopidogrel Resistance. Our reports also indicated only homozygous PONQ192R showed attenuated response to clopidogrel therapy. This results are the same as several reports [29, 30]. However, other studies could not discovery the influence of PON1 Q192R genotype on clopidogrel induced antiplatelet aggregation inhibition [31, 32], This may due to the small effects of PON1 Q192R polymorphism on platelet aggregation.

Many studies have proved that the CYP2C19*2 LOF alleles (*1/*2, *2/*2) increase the risk of MACE [33, 34]. Our study also showed that intermediated metabolizer (*1/*2) and poor metabolizers (*2/*2, *2/*3) seem to be at increased risk of adverse cardiovascular outcomes and higher rates of adverse cardiovascular events. It is similar to those studies, but Zhong et al. shed new light on no significant association between CYP2C19 polymorphisms and MACE [35]. such variation may include genetic, non-genetic, and physiologic agents among the patients. we could not find any association between MACE and PON-1 genotypes, it is in accordance with the results of Chen et al. [36] and Hulot et al. [37]. We guess that the first oxidation step for the Clopidogrel to 2-oxo-clopidogrel plays a decisive role in how much clopidogrel will enter the activation form. Therefore, the allelic mutant of CYP2C19 which play a significant role in the first oxidation step will lead to lower ADP-induced platelet aggregation inhibition rate and higher MACE. Whereas, PON-1 which is the enzyme for 2-oxo-clopidogrel transforms to the active metabolite would exert a limited effect on clopidogrel activation. This might explain the small effects of PON1 Q192R polymorphism on ADP-induced platelet aggregation inhibition rate but not MACE.

There were still some limitations in our study, Firstly, the sample size was relatively small. Secondly, the follow up duration was 12 months, a longer follow up duration is needed to confirm the results. Lastly, we did not exclude the effects of other metabolism enzymes on clopidogrel resistance. Some important markers may be missing. Therefore, our study yielded the definite conclusions.

## Conclusion

In summary, the polymorphism of CYP2C19 and PON-1 affect clopidogrel responsiveness. The polymorphism of CYP2C19 affect the adverse cardiovascular events but not PON-1. These findings.

Provided individual drug therapies among minority populations in JinHua in China.

## Data Availability

All relevant data are including in the manuscript. The datasets used and/or analyzed during the current study are available from the corresponding author upon request.

## Abbreviations

CHD: Coronary Heart Disease
DAPT: Dual antiplatelet therapy
MACE: Major adverse cardiac events
MPAR: Maximal platelet aggregation rate
PAIR: Platelet aggregation inhibition rate
PCI: Percutaneous Coronary Intervention
PON-1: Paraoxonase-1
PPP: Platelet-poor plasma
PRP: Platelet-rich plasma
